# Seronegative and seropositive rheumatoid arthritis treated with rituximab

**DOI:** 10.1186/1479-5876-10-S3-P47

**Published:** 2012-11-28

**Authors:** Maria Luisa Velloso Feijoo, Rosalia Martínez Pérez, Lucia González Mayordomo, Jose Luis Marenco de la Fuente

**Affiliations:** 1 Rheumatology Unit, Valme University Hospital, Seville, Spain

## Background

B cells play a crucial role in the pathogenesis of rheumatoid arthritis (RA). They are responsible for the autoantibodies formation such as rheumatoid factor (RF) and anti- cyclic citrullinated peptide antibodies (anti-CCP) and the production of cytokines, act as antigen presenting cells and regulate T cell functions.

Rituximab (RTX), murine monoclonal antibody which selectively targets CD20-positive B-cells, has proved to be an efective and safe therapy for active RA. Initially it was used in seropositive RA, but considering the other functions of B cells, it is logical to think that it is also useful in seronegative forms.

## Objective

To evaluate the efficacy of RTX in our series of refractory seronegative and seropositive RA.

## Materials and methods

Baseline characteristics and disease activity markers at baseline, and after 3 and 6 months of treatment with RTX (1 g x 2 weeks), were collected in 33 patients. A descriptive study was made; and the relations between variables were analyzed statistically.

## Results

The mean age was 52.06 ± 12.01 years, 75.8% female, 78.8% RF positive (26). The mean duration of illness was 7.70 ± 4.47 years. Thirty-two patientes (97%) had failed at least to one TNF antagonist.

Most of the patients (84.8% 9) received RTX with methotrexate.

The mean DAS28 at baseline was 5.7 ± 1.30; at 3 months decreased to 3.4 ± 1.22, and at 6 months to 4.15 ± 1.69 ( p < 0.0005).

At 3 months, 88.9% reached good eular response, and 63.3% at 6 months. Remission was obtained in 17.2% at 3 months and in 16.7% at 6 months.

It was also noted improvement in baseline HAQ, after 3 and 6 months (from 1.75 ± 0.767 to 0.96 ± 0.56 and 1.24 ± 0.70 respectively).

No significant differences were found between decreases in DAS 28 at 3 and 6 months compared to baseline between RF seronegative and seropositive patients, neither in good eular response, remission percentages or HAQ improvement. The data are shown in the table.

**Table 1:** 

	Mean DAS28 at baseline	Mean DAS28 at 3 months	Mean DAS28 at 6 months	Good eular response at 3 months	Good eular response at 6 months	DAS28 < 2,6 at 3 monts	DAS28<26 at 6 months	HAQ at baseline	HAQ at 3 months	HAQ at 6 months
RF +	5.73 ± 1.24	3.38 ± 1.30	4.13 ± 1.83	90.5%	65.2%	18.2%	17.4%	1.81 ± 0.77	1.00 ± 0.57	1.2 ± 0.74
RF -	5.52 ± 1.61	3.72 ± 0.93	4.22 ± 1.23	83.3%	57.1%	14.3%	14.3%	1.57 ± 0.78	0.83 ± 0.56	0.95 ± 0.54

## Discussion

The efficacy and safety of RTX has been proved in several clinical trials.

The presence of RF, low baseline functional disability and no more than one previous anti-TNF are predictors of good response to RTX, as has been recently published.

Response rates in seronegative RA, are slightly lower, although higher than placebo, as described in other publications.

In conclussion, the experience of RTX treatment in our patients with seronegative RA is positive, in terms of efficacy, due to the action on B cells and their different roles, with no significant differences comparing to seropositive RA.
